# Lanthanide sorbent based on magnetite nanoparticles functionalized with organophosphorus extractants

**DOI:** 10.1088/1468-6996/16/3/035010

**Published:** 2015-06-25

**Authors:** Carlos Basualto, José Gaete, Lorena Molina, Fernando Valenzuela, Claudia Yañez, Jose F Marco

**Affiliations:** 1 Facultad de Ciencias Químicas y Farmacéuticas/Universidad de Chile, Santos Dumont 964, Independencia, Santiago, Chile; 2 Instituto de Química-Física Rocasolano, CSIC, Calle Serrano 119, Madrid E-28006, Spain

**Keywords:** organophosphorus extractant, magnetite nanoparticles, lanthanide metal, functionalized magnetite sorbent

## Abstract

In this work, an adsorbent was prepared based on the attachment of organophosphorus acid extractants, namely, D2EHPA, CYANEX 272, and CYANEX 301, to the surface of superparamagnetic magnetite (Fe_3_O_4_) nanoparticles. The synthesized nanoparticles were coated with oleic acid, first by a chemisorption mechanism and later by the respective extractant via physical adsorption. The obtained core–shell functionalized magnetite nanoparticle composites were characterized by dynamic light scattering, scanning electron microscopy, transmission electron microscopy, thermogravimetry, infrared absorption and vibrating sample magnetometry. All the prepared nanoparticles exhibited a high saturation magnetization capacity that varied between 72 and 46 emu g^−1^ and decreased as the magnetite nanoparticle was coated with oleic acid and functionalized. The scope of this study also included adsorption tests for lanthanum, cerium, praseodymium, and neodymium and the corresponding analysis of their results. Sorption tests indicated that the functionalized nanoparticles were able to extract the four studied lanthanide metal ions, although the best extraction performance was observed when the sorbent was functionalized with CYANEX 272, which resulted in a loading capacity of approximately 12–14 mg_La_/g_MNP_. The magnetization of the synthesized nanoparticles was verified during the separation of the lanthanide-loaded sorbent from the raffinate by using a conventional magnet.

## 1. Introduction

Solvent extraction and ion exchange have been widely applied to the recovery and separation of metal ions. However, both techniques, as well as other ones in current use, present limitations when used for dilute and complex aqueous solution treatment. Conventional solvent extraction requires many stages to achieve an efficient separation and presents a limited ability of enrichment of extracted metals, given the usual organic and aqueous volume ratio employed in the process. There are also some issues resulting from the organic solvent solubility in the feed solution, the entrainment of an organic solution into an aqueous phase and crud formation because of the fine solid particles in aqueous solution, leading to significant losses of the extractants, which are commonly expensive compounds. In turn, ion exchange using solid resins presents important drawbacks related to the metal loading capacity and the desorption step.

Lanthanide elements (Ln) and their compounds, however, are reaching a level of great importance from the standpoint of their industrial applications because these elements possess various unique physical and chemical properties, which pave the way for immense possibilities for the development of many new high-tech materials for wider applications, including the preparation of functional materials. However, the chemical behavior of these elements is very similar, which makes their mutual separation difficult using conventional methods [1]. In addition, the difficulty of preparing rare-earth element compounds of high purity has been a barrier to their practical applications because the presence of even trace impurities affects their mechanical, electrical and chemical properties.

The adsorption of metal ions on natural and synthesized adsorbent bed materials has been extensively used as a method for their recovery or removal. Among these natural original materials are bentonite, chitosan, cellulose, and other vegetable raw materials. These materials are inexpensive and abundant. Among the synthesized sorbents, materials such as activated charcoal, alumina, silica, alumino-silica gels, and a broad line of metal oxides are notable. However, the main hindrance to their extensive use is the reduced surface area available for the sorption process, requiring a large amount of the sorbent material to collect a small amount of adsorbed species. Undoubtedly, good sorption performance will require the use of sorbents with the smallest particle size and highest surface area possible.

These limitations indicate the need for the synthesis or preparation of a new type of sorbents, ideally prepared using simple methods and generating sorption compounds that can be used efficiently to separate or remove metal and non-metal species normally found in dilute concentrations in aqueous solutions.

Herein lies the main aim of this study, which is the synthesis of a new type of sorbent that would enable the efficient extraction and separation of lanthanide elements from aqueous solutions and whose separation from the aqueous solution is simple and complete. Specifically, in this work, an adsorbent was prepared based on the attachment of organophosphorus acid extractants, namely, D2EHPA, CYANEX 272, and CYANEX 301, to the surface of superparamagnetic magnetite nanoparticles (MNPs). On one hand, this type of sorbent conjugate uses the organic-extractant family derived from phosphoric acid, called by the generic names of phosphoric, phosphonic, phosphinic and dithiophosphinic acids, which appears to be very promising for achieving the extraction and separation of lanthanide ions from aqueous solutions [2–8]. On the other hand, these sorbents are based on functionalized MNPs, i.e., specific molecules attached to the surface of MNPs, which have gained increasing interest because of their potential applications. The functionalization method assigns specific properties of interaction or reaction to the MNPs, whereas the magnetic attribute allows a simple separation step from the treated aqueous solution using an external magnet. Examples of their uses have included cell separation [9], magnetic resonance imaging [10], drug delivery systems [11], antibacterial activity [12], protein separation [13], and cancer treatments through hyperthermia [14], among many others.

Magnetite is the most widely used magnetic material. It is a nontoxic, inexpensive and strongly ferromagnetic compound with a Curie point of 860 K, at which temperature the effective magnetic moment is 3.9·10^−23^ J T^−1^ (4.2 *μ*_B_) [15]. It becomes superparamagnetic when the size of its domains approaches the nanoscale [16–18]. This material is an ideal candidate for use as a magnetic core that can be coated by countless molecules, yielding specific functionalities. In this article we report the synthesis of the sorbents and the characterization of the obtained core–shell functionalized magnetite nanoparticle composites. The scope of this study also includes adsorption tests for lanthanum, cerium, praseodymium, and neodymium and the corresponding analysis of the results.

## 2. Experimental details

### 2.1. Reagents

The following pure chemicals were used: FeCl_2_·4H_2_O and FeCl_3_·6H_2_O, fuming hydrochloric acid 37%, Emsure Merck; ammonia solution 25% GR for analysis, Merck; acetone GR, Merck; n-hexane absolute for analysis, Emparta Merck; and oleic acid Ph Eu Sigma-Aldrich; D2EHPA Merck, CYANEX 272 and CYANEX 301 provided by CYTEC-Chile.

### 2.2. Procedure

MNPs were precipitated from iron salts. In a 500 mL beaker, 50 mL of 0.2 M Fe^3+^ and 50 mL of 0.1 M Fe^2+^ solutions were mixed, attaining a 2:1 ratio between them. The pH was maintained at a value of 2.0, with the participant solutions degasified under an inert nitrogen atmosphere maintained during the experiments. Subsequently, the temperature was increased to 80 °C, and the pH was increased to 9 by the addition of NH_4_OH solution. The achieved mixture was allowed to stand for 30 min; the obtained precipitate was decanted using a magnet and washed with degassed distilled water. This process was repeated three times, and the obtained nanoparticles were dried and stored.

To stabilize the obtained nanoparticles, 0.65 mL of oleic acid was added; then, for the subsequent functionalization, 50 mL of a 0.03 M emulsioned organophosphorus acid extractant was added, and the mixture was then placed in an ultrasonic bath for 1 h. The emulsions were previously prepared by saponification, with the addition of NaOH for 12 h over the respective solutions of D2EHPA, CYANEX 272, or CYANEX 301 extractant dissolved in kerosene.

The resulting functionalized MNPs were separated using a magnet and washed with several portions of water with a pH of 4. Afterwards, the nanoparticles were stored in water at the same pH value for later use. In both cases the pH value of 4 was obtained by acidifying with dilute nitric acid.

Measurements of the particle size and size distribution in aqueous solution were performed using a Malvern Instruments Zetasizer Nano ZS particle analyzer. A dual FEI Inspect F50 microscope was used to record scanning electron microscopy (SEM) and transmission electron microscopy (TEM) images.

Thermogravimetric analysis (TGA) curves of the coated samples of magnetite were obtained using a NETZSCH Instruments TG209 F1 Iris thermogravimetric analyzer.

Fourier-transform infrared attenuated total reflection (FTIR-ATR) spectra were recorded using an Interspec 200X-ATR-V spectrometer (Interspectrum OU, Estonia). The measurements on dry samples were obtained using the PIKE Miracle accessory with a ZnSe crystal plate. Spectra were obtained by averaging 10 scans over the spectral range of 4000–550 cm^−1^.

Adsorption experiments were conducted in a batchwise reactor by contacting 50 mL of a 0.25 mM lanthanide metal ion feed solution with 7 mL of a concentrate of functionalized nanoparticles for 1 h while maintaining a stirring speed of 600 rpm. Then, the loaded nanoparticles were separated by magnetic decantation, and the raffinate was maintained to determine the amount of unextracted remaining metal. Lanthanide metal concentrations were measured by the Arsenazo III photometric method at 660 nm.

## 3. Results and discussion

### 3.1. Particle size determination by DLS and SEM/TEM

First, some physical analyses were performed to characterize the obtained nanoparticles. Among them, the dynamic light scattering (DLS) technique was used to determine the size distribution and average particle size for uncoated and different coated MNPs, all of them dispersed in aqueous medium.

**Figure 1.**
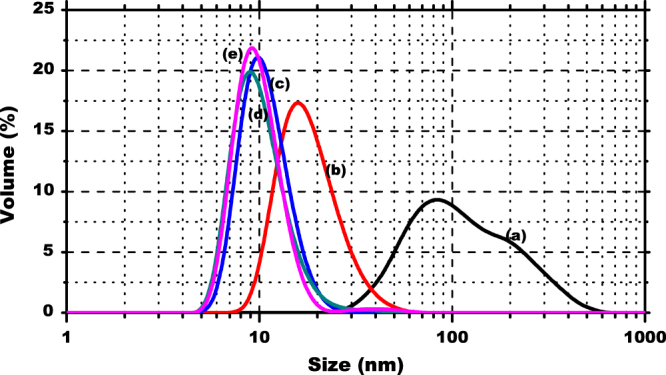
Size distribution of MNPs: uncovered (a) and stabilized with oleic acid (b), CYANEX 272 (c), D2EHPA (d) and CYANEX 301 (e).

Figure 1 shows size distributions of the initial and stabilized MNPs. Moreover, the observed analysis for the MNPs functionalized with the organophosphorus acid extractants CYANEX 272, D2EHPA, and CYANEX 301 are presented in figures 1(c)–(e), respectively.

The average diameter of the nanoparticles is listed in table 1. The results indicate that the largest diameter was obtained for the uncovered nanoparticles of magnetite. This finding appears to be contradictory because the smaller size is expected for this type of particle, without any coating; however, given that the particles are dispersed in an aqueous medium, it is highly probable that the particles tend to exhibit a high degree of agglomeration.

**Table 1.** Average size and polydispersity index for uncovered, stabilized, and functionalized magnetite nanoparticles.

Magnetite nanoparticles	Average size (nm)	PI
Uncovered MNP	134.0	0.2
Oleic acid stabilized MNP	18.8	0.2
CYANEX 272 functionalized MNP	10.9	0.3
D2EHPA functionalized MNP	10.5	0.3
CYANEX 301 functionalized MNP	10.4	0.3

The polydispersity index (PI) varies from 0 to 1. PI < 0.1 indicates a high homogeneity in the particle population, whereas high PI values suggest a broad size distribution or even several populations [19]. The obtained values of PI for these coated and functionalized MNPs are between 0.2 and 0.3, approximately, which indicates the particles are sufficiently homogeneous sizes. Evidently, it was expected that the functionalized nanoparticles would be larger than the stabilized nanoparticles; however, in this case, it appears size contraction occurs when the extractant is added to the stabilized nanoparticles with oleic acid.

**Figure 2.**
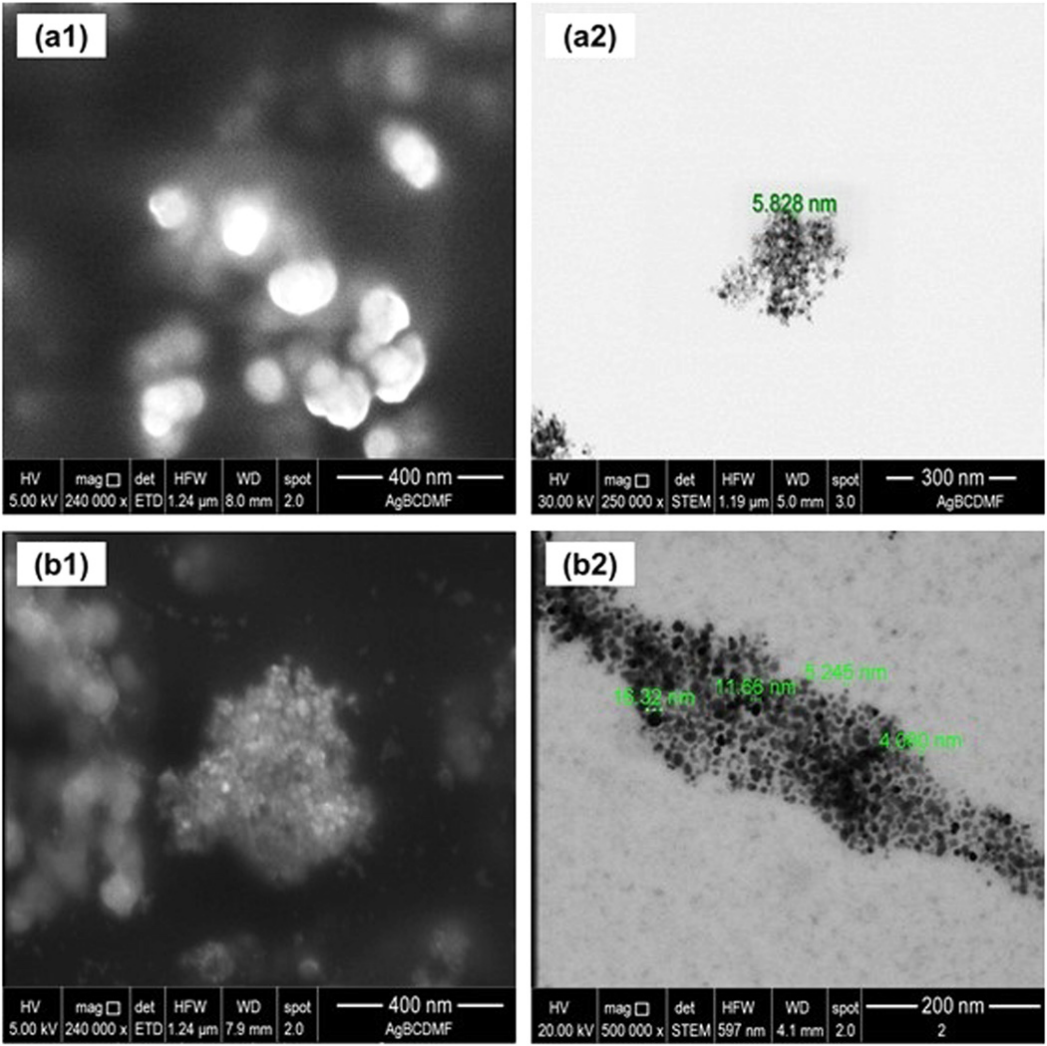
SEM (1) and TEM (2) images of uncovered MNPs (a1), (a2) and oleic acid stabilized MNPs (b1), (b2).

**Figure 3.**
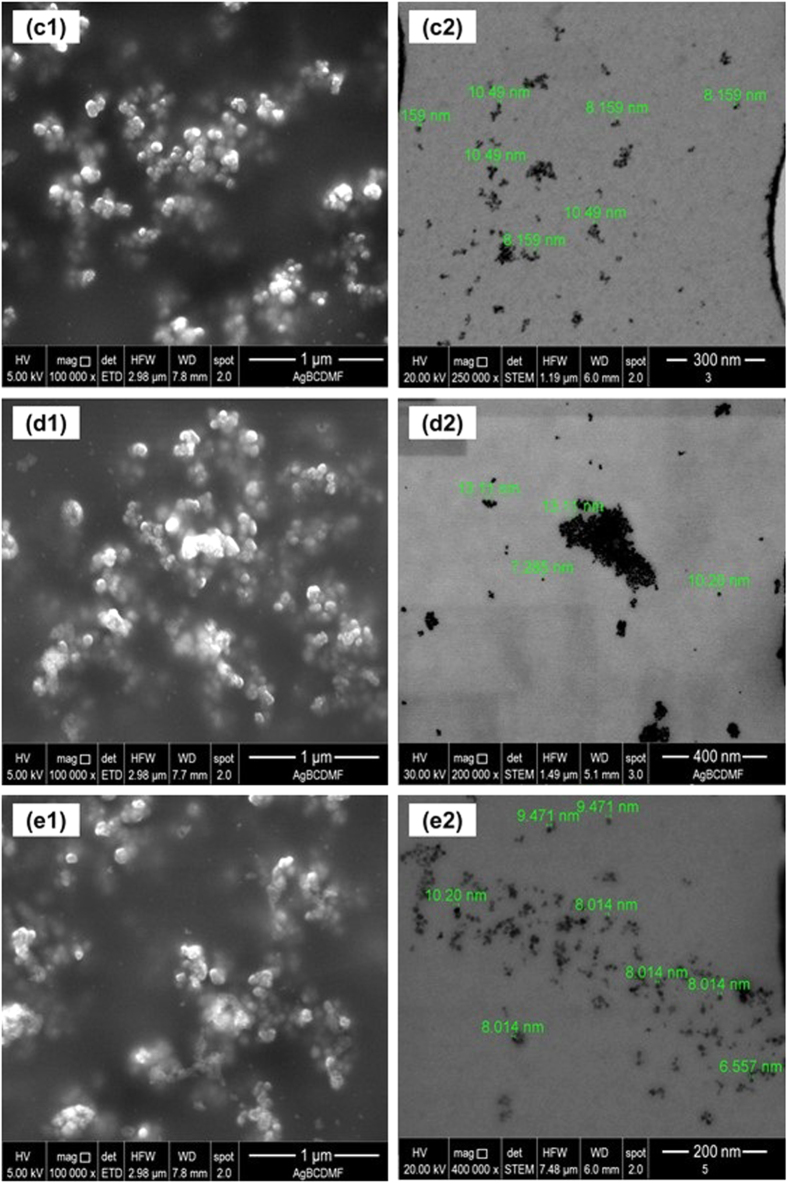
Images of MNP functionalized with (a) CYANEX 272, (b) D2EHPA and (c) CYANEX 301. The numbers (1) and (2) indicate SEM and TEM images, respectively.

SEM and TEM are used for analyzing particle properties such as their size, shape and texture. Whereas SEM allows investigation of the surface appearance, TEM allows examination inside the internal structure of the material. The SEM and TEM measurement results of the nanoparticles in their various developmental stages are presented in figures 2 and 3.

The particles exhibit significant agglomeration, as revealed by SEM and TEM images. This agglomeration effect would occur when these particles are suspended in aqueous solution too.

The TEM images confirm that despite being agglomerated, the nanoparticles do not lose their individuality consisting of a core smaller than 10 nm and an organic coating. Moreover, the extractants attached to the surface of the MNPs form a coating, which gives them the same size, shape, and appearance. When DLS measurements of the average size are correlated with the results obtained through SEM and TEM micrographs, the effect of agglomeration resulting from the different coatings of nanoparticles remains clear when they are dispersed in an aqueous system.

### 3.2. Thermogravimetric analysis

**Figure 4.**
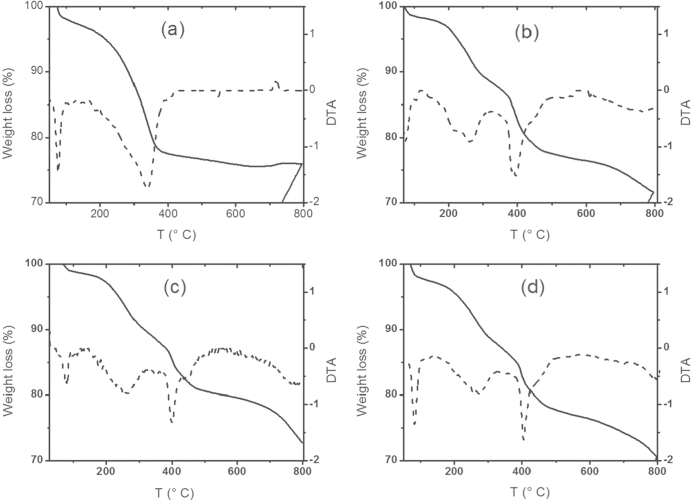
TGA (solid lines) and DTA (dashed lines) curves for (a) MNP, (b) MNP-Cy 272, (c) MNP-Cy 301 and (d) MNP-D2EHPA.

Using this technique, it is possible to determine the structure and/or composition of functionalized nanoparticles through the mass loss with temperature. The samples were subjected to an increasing temperature program; the results indicate whether the particles underwent a desorption, sublimation, or decomposition process, among others. TGA and differential thermal analysis (DTA) curves were obtained for the different synthesized nanoparticles, which are presented in figure 4.

The TGA curves presented in figure 4, represented by solid lines, reveal that non-uniform mass losses are observed as the temperature increases, indicating that the mass loss is likely due to the release of typical gases by decomposition, first of organic material associated with the physical sorption of the extractant onto the nanoparticle and then with the chemisorption of the oleic acid.

A summary of the information obtained from the TGA and DTA curves of all the coated nanoparticles is presented in table 2.

**Table 2.** Relationship between the lost weight of the synthesized nanoparticles and temperature obtained from the TGA and DTA curves.

Sample	Temperature	Weight loss%
Stabilized NPM	338 °C	18
NPM-Cy 272	260 °C	8
	383 °C	12
NPM-Cy 301	264 °C	8
	446 °C	11
NPM- D2EHPA	248 °C	10
	397 °C	11

In all the TGA curves presented in figure 4, a weight loss of about 1% is observed below 100 °C, which can be assigned to any remaining volatile solvent used in the synthesis process. In curve (a) corresponding to the stabilized NPM, a significant weight loss is observed at 338 °C, which is due to the desorption and decomposition of the chemisorbed oleate group. This same lost weight is observed at temperatures between 383 and 446 °C in the curves of the nanoparticles functionalized with the extractants: (b) Cyanex 272, (c) and Cyanex 301 and (d) D2EHPA. However, for these functionalized nanoparticles, an additional lost weight between 248 and 260 °C is observed, which can be attributed to the physical desorption and subsequent decomposition of the respective extractants. Thus, in conclusion, using TGA, it is possible to determine that the MNPs are effectively coated by an initial layer of oleic acid and by a second layer of the respective extractant.

### 3.3. FTIR-ATR spectroscopy analysis

**Figure 5.**
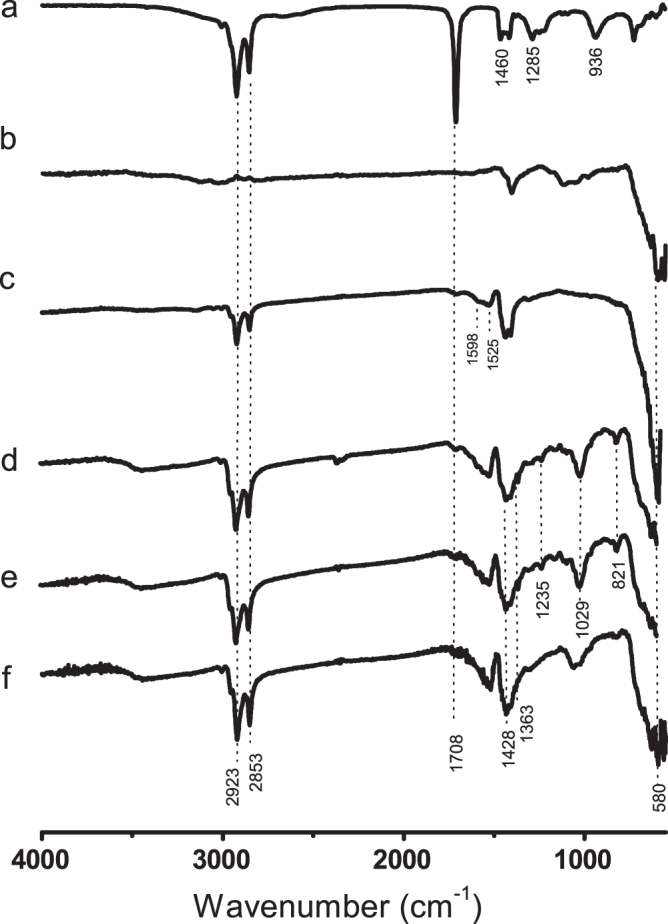
FTIR-ATR spectra of (a) oleic acid, (b) MNP, (c) oleic acid stabilized MNP, (d) MNP-CYANEX 272, (e) MNP-CYANEX 301 and (f) MNP- D2EHPA.

FTIR-ATR is an analytical technique that provides an improved infrared spectrum of the sample. It allows us to verify the presence of characteristic chemical bonds in many species to confirm their attachment onto the surface of nanoparticles, forming a shell-type structure. The obtained spectra for oleic acid, MNP, stabilized MNP and the different functionalized MNPs are presented in figure 5.

Figure 5(a) shows the typical bands observed for pure oleic acid: two sharp bands at 2923 and 2853 cm^−1^ attributed to asymmetric and symmetric CH_2_ stretching, respectively. The band corresponding to the C=O stretching appears at 1708 cm^−1^, whereas the band at 1285 cm^−1^ is attributed to C-O stretching. The bands appearing at 1460 and 936 cm^−1^ correspond to in-plane and out-of-plane O–H groups, respectively [20]. The spectrum of magnetite (figure 5(b)) contains an Fe–O band at 580–600 cm^−1^, which was also observed for all the coated samples of MNPs (figures 5(c)–(f)).

**Figure 6.**
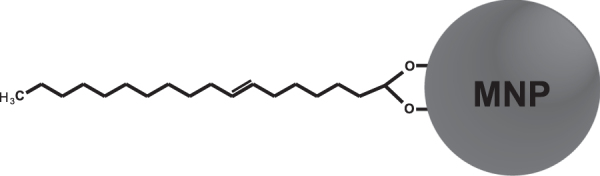
Oleic acid chemically attached to magnetite nanoparticle surface.

The FTIR spectrum recorded from the oleic acid–coated MNP (figure 5(c)) clearly shows the disappearance of the carbonyl group signal, whereas the doublet characteristic of the methylene groups of oleic acid remains. Hence, the results indicate that oleic acid is attached at the surface of the nanoparticles, as depicted in figure 6. The broad bands at approximately 1598 and 1525 cm^−1^ can be assigned to asymmetric and symmetric COO– stretching, respectively, which indicates that oleic acid is chemically attached as bidentate chelate R–COO– to the magnetite nanoparticles. This covalent interaction was reported previously [20], with the bands of COO– stretching appearing at slightly higher frequencies.

The FTIR spectra of the Cyanex 272, D2EHPA and Cyanex 301 functionalized MNPs (figures 5(d)–(f)) show the characteristic peaks: the bands at 2953 and 2850 cm^−1^ can be assigned to –CH_2_ stretching, and the two peaks appearing at 1428 and 1363 cm^−1^ can be assigned to –CH deformation. The peak observed for Cyanex 272 and D2EHPA at 1160 cm^−1^ corresponds to the P = O double bond, and the band at 821 cm^−1^ is attributed to C–O–P stretching [21]. The P–O vibration and stretching bands are observed at 1235 and 1024 cm^−1^, respectively.

The absorption bands at 711, 731 and 805 cm^−1^ of Cyanex 301 assigned to the S = P vibrations [22] cannot be observed in the spectrum.

### 3.4. Magnetism properties

The measurements of the magnetic properties using a vibrating sample magnetometer (VSM) are necessary for determining the magnetic capacity of the MNPs, including both the uncoated magnetite and those coated using the organic compounds.

According to the literature, the best magnetic behavior for magnetite particles is obtained when the smaller particles are synthesized, given that in that case, the particles would be constituted by magnetic monodomains, specifically in the range of a few nanometers [23, 24], with no coercivity behavior because under these conditions, they are superparamagnetic. Optimum conditions are required for magnetite precipitation, among them a final pH of 9, which assures the synthesis of a nanoparticulate material with superior magnetic properties [16–18, 22].

Once the MNPs were coated with the extractants, the nanoparticles were used in the adsorption step of the lanthanide elements contained in the aqueous solution. Because the main purpose of preparing these nanoparticles was to facilitate the separation of the lanthanide-loaded sorbent from the aqueous raffinate, the nanoparticles should respond optimally when an external magnetic field is applied.

**Figure 7.**
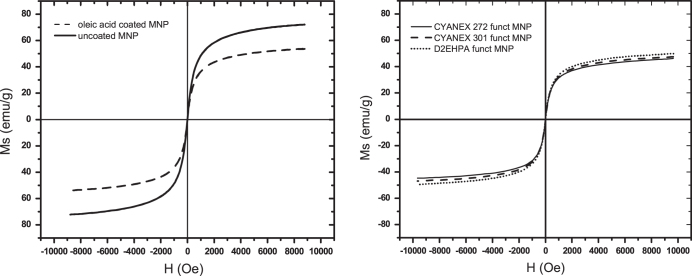
Magnetic hysteresis loops for (a) uncoated and oleic acid-coated MNP and (b) CYANEX 272, CYANEX 301 and D2EHPA functionalized MNP.

Figure 7 shows the magnetic responses, in emu/g units, when an external magnetic field (H) in Oersted units was applied to the uncoated and oleic acid-coated nanoparticles (a) and to the functionalized nanoparticles of magnetite (b).

As observed in figure 7, the obtained curves demonstrate that all the nanoparticles are constituted by a single magnetic domain with no significant coercivity, which confirms that all of the nanoparticles are superparamagnetic. Furthermore, greater coating thicknesses of the nanoparticles result in smaller magnetic responses against an external magnetic field.

The saturation magnetization capacity decreasea from 72 emu g^−1^ to 55 emu g^−1^ for the oleic acid-stabilized particles to 46–49 emu g^−1^ for the phosphoric extractant–functionalized MNPs. However, the obtained magnetization value for those functionalized nanoparticles was sufficient to separate the lanthanide-loaded MNP from the raffinate using a simple magnet device.

Based on all these characterization results, it is possible to conclude from the SEM and TEM analyses that the functionalized and coated nanoparticles are effectively constituted by individual magnetic domains of approximately 10 nm, which are dispersed according to the DLS results. Moreover, it has been demonstrated that the functionalized MNPs consist of a core of magnetite with an organic coating shell. In addition, the FTIR-ATR technique confirmed the attachment of organophosphorus extractants to the surface of the nanoparticles.

### 3.5. Adsorption behavior

**Figure 8.**
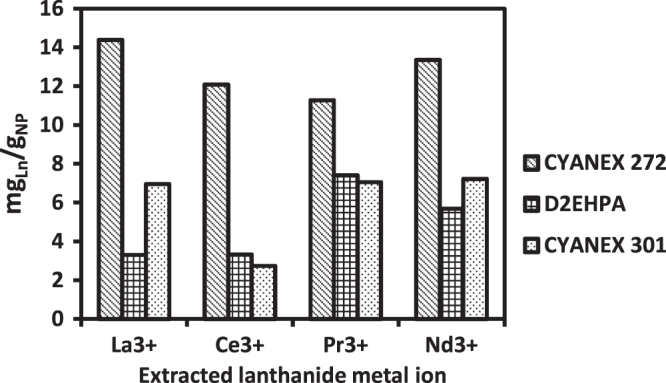
Loading capacities of functionalized magnetite nanoparticles for lanthanum, cerium, praseodymium, and neodymium.

With the objective of testing the feasibility of adsorbing lanthanide elements present in an aqueous solution onto these functionalized MNPs, some adsorption experiments were conducted. Specifically, the adsorption of four lanthanide ions (La^3+^, Ce^3+^, Pr^3+^ and Nd^3+^) was studied. Adsorption runs were performed using the same suitable experimental conditions employed previously in a liquid–liquid extraction study, namely, an initial pH of 4 and a content of all the tested lanthanides of 0.25 mM. The results are presented in figure 8, where the lanthanide loading capacity is expressed in mg lanthanide per gram of sorbent. The results for the three studied phosphoric extractants are compared in the same figure.

These first exploratory results clearly indicate that MNPs functionalized with CYANEX 272 extractant exhibit better sorption ability, practically double that observed for the D2EHPA and CYANEX 301 extractants. The functionalized nanoparticles exhibited no significant selectivity for a given lanthanide element, which is consistent with the similar chemical activities of all these lanthanides. As can be observed in figure 8, the CYANEX 272 loading capacity varied between 12 and 14 mg_La_/g_MNP_.

Other groups reported higher adsorption capacities for lanthanide extraction by different sorbent materials: Liao *et al* 70 mg_La_/g_NP_ for citrate coated magnetic nanoparticles [25]; Kondo *et al* 230 mg_La_/g_MC_ using microcapsules containing 2-ethylhexylphosphonic acid mono-2-ethylhexyl ester [26]; Wu *et al* 55.9 mg_La_/g_NP_ using 2-ethylhexyl phosphonic acid mono-2-ethylhexyl ester-grafted magnetic silica nanocomposites [27]; Awwad *et al* found 175.4 mg_La_/g and 250 mg_Er_/g of activated carbon [28]; Dupont *et al* have found in the literature several values for gadolinium uptake, such as 14 mg_Gd_/g_NP_ of Fe_3_O_4_, 20 mg_Gd_/g_NP_ of SiO_2_, 41 mg_Gd_/g_NP_ of TiO_2_, and 113 mg_Gd_/g_NP_ of Fe_3_O_4_ (TMS-EDTA), among others [29].

Although these particles have higher adsorption capacities than the functionalized magnetite presented in this work, their density should be considered—magnetite is heavier, and therefore its surface area per gram of sorbent is smaller.

## 4. Conclusions

A new type of sorbent was synthesized and tested to extract some lanthanide elements from aqueous solutions. MNPs functionalized with three organophosphorus extractants (CYANEX 272, D2EHPA, and CYANEX 301) were prepared, resulting in nanoparticles with an average size in the range of 10 nm. This particle size was verified using DLS and SEM/TEM techniques. In addition, TGA and FTIR-ATR techniques demonstrated that the organophosphorus extractants were attached to the stabilized nanoparticles, forming a coating shell around them.

All the prepared nanoparticles exhibited a high saturation magnetization capacity, which varied between 72 and 55 emu g^−1^ to 46 emu g^−1^ and decreased as the MNP was coated with oleic acid and functionalized with the organophosphorus extractants. Sorption tests indicated that the functionalized MNPs were able to extract the four studied lanthanide metal ions, although the best extraction performance was observed for CYANEX 272, which exhibited a loading capacity of approximately 12–14 mg_La_/g_MNP_. The magnetization of the synthesized nanoparticles was verified during the separation of the lanthanide-loaded sorbent from the raffinate, where it was possible to collect all the nanoparticles onto a conventional magnet in a simple step.
